# Lower serum expression of miR-181c-5p is associated with increased plasma levels of amyloid-beta 1–40 and cerebral vulnerability in normal aging

**DOI:** 10.1186/s40035-019-0174-8

**Published:** 2019-11-04

**Authors:** Marta Manzano-Crespo, Mercedes Atienza, Jose L. Cantero

**Affiliations:** 1 Laboratory of Functional Neuroscience, Pablo de Olavide University, Ctra. de Utrera Km 1, 41013 Seville, Spain; 2 CIBERNED, Network Center for Biomedical Research in Neurodegenerative Diseases, 28031, Madrid, Spain

**Keywords:** Aging, Alzheimer’s disease, Blood biomarkers, microRNAs, Amyloid-beta, FDG-PET, Entorhinal cortex

## Abstract

**Background:**

Previous studies have shown that expression levels of miR-181c are downregulated by amyloid-β (Aβ) deposition and chronic cerebral hypoperfusion, both factors largely associated with the development of AD. Moreover, reduced 2-[18F]fluoro-2-deoxy-D-glucose (FDG)-PET brain metabolism and volume loss of regions of the medial temporal lobe have been generally recognized as hallmarks of AD. Based on this evidence, we have here investigated potential associations between serum levels of miR-181c-5p and these AD signatures in asymptomatic elderly subjects.

**Methods:**

Ninety-five normal elderly subjects underwent clinical, cognitive, structural MRI, and FDG-PET explorations. Serum expression levels of miR-181c-5p and plasma Aβ concentrations were further analyzed in this cohort. Regression analyses were performed to assess associations between serum miR-181c-5p levels and cognitive functioning, plasma Aβ, structural and metabolic brain changes.

**Results:**

Decreased serum expression of miR-181c-5p was associated with increased plasma levels of Aβ_1–40_, deficits in cortical glucose metabolism, and volume reduction of the entorhinal cortex. No significant associations were found between lower miR-181c-5p levels and cognitive deficits or cortical thinning.

**Conclusions:**

These findings suggest that deregulation of serum miR-181c-5p may indicate cerebral vulnerability in late life.

## Background

Aging is an extremely complex and inexorable process characterized by gradual accumulation of biological damage that leads to impaired cell homeostasis, decreased organ mass, and loss of functional reserve of the body’s systems [1]. Aging is not exactly a disease but has the capacity to increase the risk for developing a wide range of chronic non-communicable conditions [2]. Most importantly, experimental manipulations aimed at slowing down aging have shown to improve survival, delay disease onset, and reduce the rate of aging-related chronic disorders [3–5], suggesting that intervening in aging could prevent chronic diseases and extend healthspan [6, 7].

With the unprecedented aging population, the burden of dementia has increased substantially. Alzheimer’s disease (AD), the most common cause of dementia among older people, is rapidly becoming a major public health problem in developed countries. Identifying AD vulnerability in asymptomatic individuals is a challenging endeavor due to the remarkable variability in aging phenotypes, lifestyles, and environmental exposures. Considering that AD is becoming a healthcare burden of epidemic proportion, there is an urgent need for identifying biomarkers of susceptibility to developing AD before cognitive symptoms arise. Ideally, these biomarkers should be minimally invasive and cost-effective, uncover aspects of AD pathology, and correlate with subclinical changes in AD-related brain regions.

MicroRNAs (miRNAs) are small, non-coding RNAs that regulate gene expression and protein synthesis by inducing degradation or suppressing translation of the target messenger RNA (mRNA) [8]. Evidence suggests that miRNAs are released from neurons into the bloodstream, where they are highly stable over time and can be detected non-invasively [9, 10]. Consequently, blood miRNA expression levels may reveal dysregulation of brain-enriched miRNAs due to different aspects of AD pathogenesis, including inflammation, lipid metabolism, oxidative stress, and proteinopathy [11]. Many recent studies have identified blood miRNA expression profiles in AD patients, suggesting that they are able to differentiate clinical AD from normal aging with reasonable accuracy [12–18]. However, there is a lack of research on AD-related miRNAs combined with neuroimaging markers in asymptomatic individuals, who may be selected for timely interventions to reduce the risk of developing dementia.

Previous studies have revealed that miR-181c regulates amyloid-β (Aβ) deposition through changes in membrane ceramide levels and lipid rafts [19]. Expression of miR-181c, especially miR-181c-5p, has found to be correlated with increased Aβ levels [20], and deregulated in brain [21–23] and blood of AD patients [12, 17, 24, 25]. Based on this evidence, we have here investigated potential associations between serum expression levels of miR-181c-5p and plasma Aβ, cognitive and brain changes in asymptomatic elderly subjects. Our hypothesis is that lower levels of miR-181c-5p should parallel changes in plasma Aβ concentrations, deficits in AD-related brain regions, and impaired cognition, likely revealing greater cerebral vulnerability in late life.

## Methods

### Participants

Ninety-five normal elderly subjects, recruited from senior citizens associations, health screening programs, and hospital outpatient services, participated in the study. They showed normal cognitive performance in the neuropsychological tests relative to appropriate reference values for age and education level. Individuals with medical conditions and/or history of conditions that may affect brain structure or function (e.g., neurodegenerative diseases, stroke, head trauma, hydrocephalus, and/or intracranial mass) were not included in the study. All subjects showed a global score of 0 (no dementia) in the Clinical Dementia Rating (CDR), normal global cognitive status in the Mini Mental State Examination (MMSE) (scores ≥26), and normal independent function –assessed by the Spanish version of the Interview for Deterioration in Daily Living Activities [26]. Depression was excluded (scores ≤5) by the Geriatric Depression Scale [27]. All participants gave informed consent to the experimental protocol approved by the Ethical Committee for Human Research at the University Pablo de Olavide according to the principles outlined in the Declaration of Helsinki.

### Neuropsychological assessment

A neuropsychological battery covering memory, executive functioning and language was administered to all participants. Subjective memory was evaluated with the Memory Functioning Questionnaire (MFQ), while objective memory was assessed with the Free and Cued Selective Reminding Test (FCSRT). The Tower of London (TOL) and the Boston Naming Test (BNT) were administered to evaluate executive function and naming, respectively.

### Total RNA isolation and miRNA quantification by qRT-PCR

Venous blood samples were collected in serum-gel 9 ml tubes (Sarstedt S-Monovette®) after overnight fasting, centrifuged at 1900 g for 10 min (4 °C) followed by a second centrifugation at 16000 g for 5 min (4 °C), and stored at − 80 °C. Total RNA was isolated from 200 μl aliquots of serum in each sample using the miRNeasy Serum/Plasma kit (Qiagen, Germany). Synthetic miRNA cel-miR-39-3p (3.5 μl, 1.6 × 10^8^ copies/μl) was used for sample-to-sample normalization in RNA isolation. Both concentration (range: 8–25 ng/μl) and purity (260/280 ratio: 1.5–2) of RNA were measured using the NanoDrop 2000 spectrophotometer (Thermo Fisher Scientific), and RNA samples were stored at − 80 °C until use.

RNA was reverse transcribed with the TaqMan® miRNA Reverse Transcription Kit and miRNA-specific stem-loop primers (Applied Biosystems), according to the manufacturer’s instructions. The reverse transcription (RT) reaction (15 μl) was composed of 5 μl of RNA (at a concentration of 10 ng/μl), 7 μl of master mix, and 3 μl of miRNA-specific stem-loop RT primer. Reactions were performed in triplicate for the miR-181c-5p, incubated at 16 °C for 30 min, 42 °C for 30 min, 85 °C for 5 min, and then maintained at 4 °C. The complementary DNA (cDNA) products were stored at − 80 °C until further analysis.

For quantitative real-time PCR (qRT-PCR), the reaction (20 μl) for each sample was composed of 1.33 μl of cDNA products, 1 μl of TaqMan® Small RNA Assay (Applied Biosystems), 10 μl of TaqMan® Universal PCR Master Mix II (2X), and 7.67 μl of nuclease-free water. Each PCR reaction was performed in triplicate with a StepOnePlus real-time PCR system (Applied Biosystems) on a 96-well plate, and incubated at 4 °C for 2 min, 95° for 10 min, followed by 40 cycles at 95 °C for 15 s and 60 °C for 1 min. Before analysis, individual cycle threshold (Ct) values were examined to detect outliers. Replicas showing Ct differences higher than 0.5 were systematically repeated. Validated Ct values were averaged and normalized to the mean of the spiked cel-miR-39-3p in each sample (Additional file 1: Figure S1). The relative expression of miR-181c-5p was calculated with the fold change method [28], and used for statistical purposes. We measured the amplification efficiency of the qPCR reactions based on the slope of the standard curve. PCR efficiency was 97% (for cel-miR-39-3p) and 96% (for miR-181c-5p). TaqMan® miRNA Assays (hsa-miR-181c-5p and cel-miR-39-3p) for qRT-PCR experiments were purchased from Applied Biosystems. RNA isolation and miRNA quantification methods have been reported elsewhere [29].

### Plasma Aβ levels

Blood samples for miRNA quantification and Aβ analysis were collected at the same time in all participants. Plasma Aβ levels were determined by a double-antibody sandwich ELISA (human Aβ_1–40_ and high sensitive Aβ_1–42_, Wako Chemicals, Tokyo, Japan). Briefly, venous blood samples were collected after overnight fasting in 10 mL K2-ethylenediaminetetraacetic acid (EDTA) coated tubes (BD Diagnostics), and immediately centrifuged (1989 g) at 4 °C for 5 min. Supernatant plasma was collected into polypropylene tubes containing 250 μL of plasma mixed with 8.32 μL of a protease inhibitor cocktail (cOmplete Ultra Tablets mini, Roche). Plasma samples were stored at − 80 °C and thawed immediately before assay.

Samples and standards were incubated overnight at 8 °C with antibodies specific for Aβ_1–40_ or Aβ_1–42_ peptides, and the wells were read for absorption at 450 nm on a Victor 3 system (PerkinElmer, Waltham, MA), according to the manufacturer’s instructions. Plasma Aβ levels were measured in duplicate (50 μL), and the average of the two measurements (pg/ml) was used for statistical purposes. Both inter-assay and intra-assay coefficients of variation were below 10%. The detection limit for these assays was 1.04 pg/ml for Aβ_1–40_ and 0.54 pg/ml for Aβ_1–42_.

### MRI and FDG-PET acquisition

Structural brain images were acquired on a Philips Achieva 3 T MRI scanner equipped with a body transmit coil and an 8-channel receive head coil (Philips, Best, Netherlands). T1-weighted magnetization-prepared rapid gradient echo (MP-RAGE) cerebral images were obtained for each participant. Acquisition parameters were empirically optimized for gray/white matter contrast (repetition time = 2300 ms, echo time = 4.5 ms, flip angle = 8°, matrix dimensions = 320 × 320, voxel size = 0.8 mm isotropic resolution, no gap between slices, acquisition time = 9.1 min). Head motion was controlled using a head restraint system and foam padding around the subject’s head.

FDG-PET brain images were acquired on a whole-body PET-TAC Siemens Biograph 16 HiREZ scanner (Siemens Medical Systems, Germany). Subjects fasted for at least 8 h before PET examination, and they were scanned at the same time of the day (8:00–9:00 am). Intravenous lines were placed 10–15 min before tracer injection of a mean dose of 370 MBq of 2-[18F]fluoro-2-deoxy-D-glucose (FDG). PET scans lasted approximately 30 min. All PET images were corrected for attenuation, scatter and decay, smoothed for uniform resolution, and reconstructed with 2.6 × 2.6 × 2 mm voxel resolution using back-projection filters. MRI-based correction of FDG-PET data for partial volume effects was performed with the PMOD software v3.208 (PMOD Technologies Ltd., Switzerland) using the Müller-Gartner approach.

### Estimation of surface-based cortical thickness and cortical glucose metabolism

MRI data were processed using the analysis pipeline of Freesurfer v6.0 (https://surfer.nmr.mgh.harvard.edu/) that involves intensity normalization, registration to Talairach, skull stripping, white matter (WM) segmentation, tessellation of the WM boundary, and automatic correction of topological defects [30]. Pial surface misplacements and erroneous WM segmentation were manually corrected on a slice-by-slice basis to enhance the reliability of cortical thickness measurements. Cortical thickness maps were smoothed using non-linear spherical wavelet-based de-noising schemes [31].

To map the FDG uptake onto cortical surfaces, we first co-registered individual FDG-PET images to T1-weighted images using PMOD tools. Next, partial volume corrected cortical FDG-PET images were sampled onto the subject’s cortical surface, transformed to the Freesurfer standard surface space, and smoothed with non-linear spherical wavelet-based de-noising schemes [31]. Finally, FDG activity assigned to each cortical surface vertex was normalized by the FDG activity of the entire cortex using an iterative vertex-based statistical method that excludes group-dependent vertices from calculation of global activity [32].

Anatomical ROIs of AD-related regions were created using a semiautomatic approach implemented in Freesurfer. Briefly, segmented brain images were parcellated into different brain regions according to the Destrieux atlas [33] and the Freesurfer automatic subcortical segmentation (aseg), respectively. This allowed us to obtain volume measurements of hippocampus, entorhinal cortex and parahippocampal gyrus, regions that have shown to be affected at early stages of AD [34].

### Statistical analyses

We first assessed whether serum expression of miR-181c-5p, plasma Aβ, and cognitive scores deviated from normality by applying the Kolmogorov-Smirnov test with the Lilliefors correction. Next, linear regression analyses were conducted to evaluate whether miR-181c-5p was associated with Aβ levels (Aβ_1–40_ and Aβ_1–42_, separately) and/or cognitive performance. Regression analyses were adjusted by age, sex and/or ApoE4 if these factors showed an statistically significant effect on any of the dependent variables. The alternative Aβ peptide was included as a confounding factor to mitigate its potential influence on the relationship between the Aβ peptide of interest and miR-181c-5p levels. We also investigated the impact of sex on regression coefficients by including the product of the dummy variable for sex and the miR-181c-5p as an interaction term in the regression model. These analyses were performed with SPSS v22 (SPSS Inc. Chicago, IL).

Vertex-wise linear regression analyses adjusted by age, sex and/or ApoE4 were further performed to determine whether serum expression levels of miR-181c-5p were associated with variations in cortical thickness/cortical glucose consumption. Results were corrected for multiple comparisons using a previously validated hierarchical statistical model [35]. This procedure first controls the family-wise error rate at the level of cluster by applying random field theory over smoothed statistical maps; and next controls the false discovery rate within significant clusters at the level of vertices over unsmoothed statistical maps. A significant cluster was defined as a contiguous set of cortical surface vertices that met the statistical threshold criteria (*p* < 0.05 after correction for multiple comparisons) and whose surface area was greater than 40 mm^2^.

Finally, linear regression analyses, adjusted by intracranial volume (ICV), as well as by age, sex and/or ApoE4 if necessary, were conducted to evaluate whether serum expression levels of miR-181c-5p were correlated with volume changes in the hippocampus, entorhinal cortex and/or parahippocampal gyrus. Regression analyses were also conducted for women and men separately. If at least one of the two groups showed significant results, we evaluated the effect of sex on regressions coefficients.

## Results

### Relationship between serum miR-181c-5p, plasma Aβ levels, and cognitive performance

**Fig. 1:**
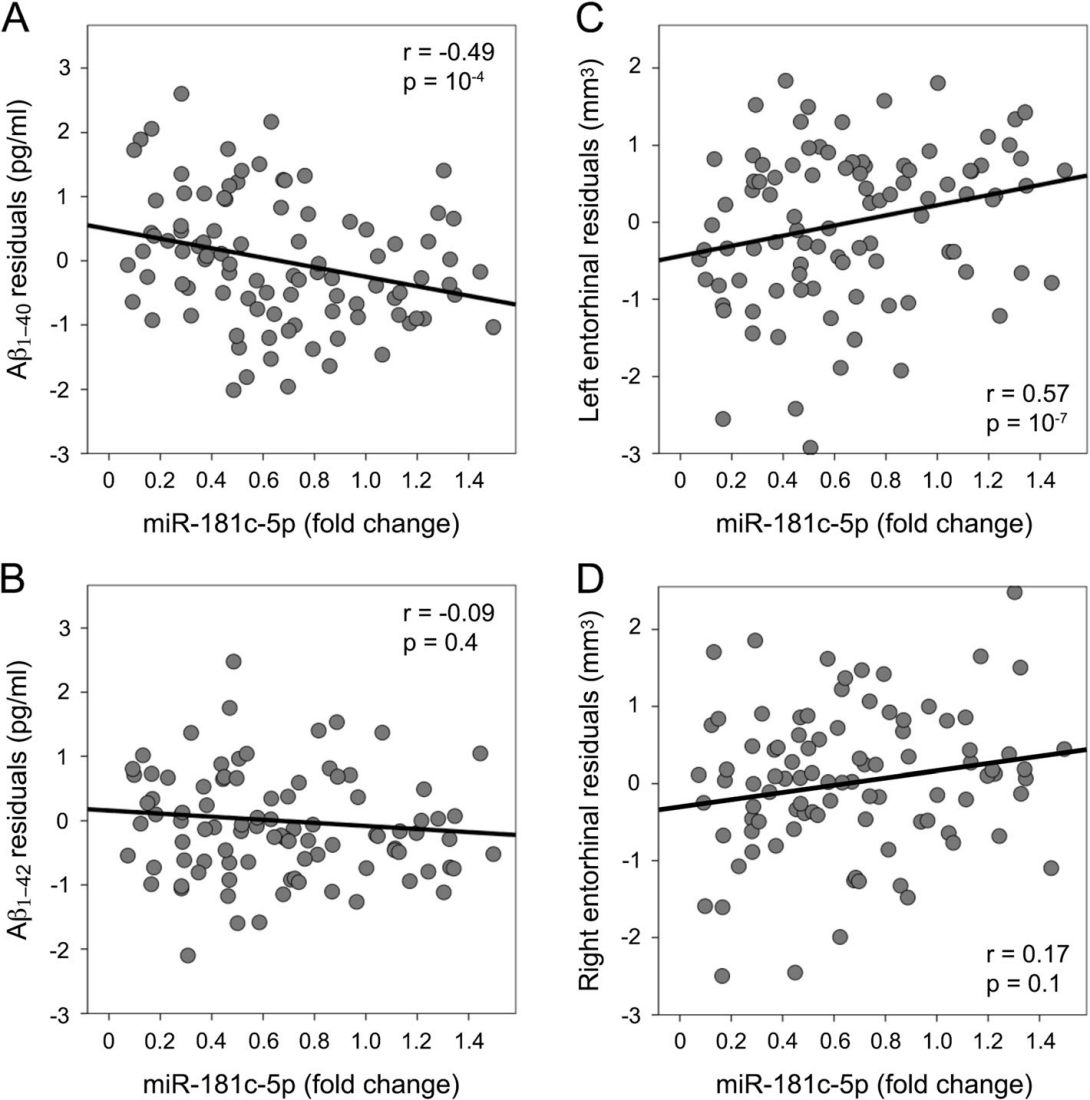
Correlations between serum levels of miR-181c-5p and plasma Aβ levels (**a** and **b**), and volume of the entorhinal cortex (**c** and **d**). Variables included in the scatter plots correspond to the standardized residuals obtained from linear regression analyses adjusted by age, Aβ_1–42_ (in the case of Aβ_1–40_), Aβ_1–40_ (in the case of Aβ_1–42_), and ICV (in the case of left and right entorhinal cortex). Note that only correlations with Aβ_1–40_ and left entorhinal cortex yielded significance

Serum miR-181c-5p, Aβ levels and cognitive scores were normally distributed, allowing the use of parametric statistical tests. Table 1 shows demographic, neuropsychological, blood, and cerebral markers of the study sample. While cognitive performance was not associated with miR-181c-5p levels, regression analyses adjusted by age and Aβ_1–42_ levels revealed that lower levels of miR-181c-5p were significantly correlated with higher Aβ_1–40_ (*r* = − 0.49, F_3,91_ = 9.52, *p* = 10^− 4^) (Fig. 1a), but not with Aβ_1–42_ concentrations (Fig. 1b). When these analyses were segregated by sex (Additional file 2: Figure S2A-B), Aβ_1–40_ was related to mir-181c-5p levels only in women (*r* = − 0.37, F_3,37_ = 4.51, *p* = 10^− 2^). However, the regression coefficients did not significantly differ between women and men.

**Table 1:** Demographic, neuropsychological, blood, and cerebral markers of the study sample

	Sample (*N* = 95)
Age	68.8 ± 4 (62–78)
Sex (M/W)	54/41
ApoE ε4 (yes/no)	21/74
CDR	0
MMSE	29.3 ± 1.2 (26–30)
MFQ	36.8 ± 10.5 (16–66)
FCSRT	13.7 ± 2 (8–16)
TOL	413 ± 139 (132–824)
Boston naming test	52.3 ± 4.5 (40–59)
miR-181c-5p (fold change)	0.66 ± 0.37 (0.07–1.5)
Aβ_1–40_ (pg/ml)	228.8 ± 32.6 (167.7–316)
Aβ_1–42_ (pg/ml)	24 ± 7.4 (7.6–59)
L hippocampus (mm^3^)	3140 ± 328 (2450–4043)
R hippocampus (mm^3^)	3242 ± 330 (2487–4080)
L entorhinal (mm^3^)	1144 ± 124 (862–1499)
R entorhinal (mm^3^)	1670 ± 199 (1131–2162)
L parahippocampal (mm^3^)	3355 ± 635 (2046–5411)
R parahippocampal (mm^3^)	3154 ± 539 (2090–4488)

Results are expressed as mean ± standard deviation for each group, range (min-max)

*M* Men, *W* Women, *CDR* Clinical dementia rating, *MMSE* Mini mental state examination, *MFQ* Memory functioning questionnaire, *FCSRT* Free and cued selective reminding test, *TOL* Tower of London, *L* Left and *R* Right

### Relationship between serum miR-181c-5p levels and volume of AD-related brain regions

Table 1 contains mean volume of hippocampus, entorhinal cortex and parahippocampal gyrus of the study sample. Regression analyses adjusted by age and ICV showed that decreased serum levels of miR-181c-5p were significantly correlated with volume reduction of the entorhinal cortex (*r* = 0.59, F_3,91_ = 16, *p* = 10^− 7^). This relationship was mainly evident for the left entorhinal cortex (*r* = 0.57, F_3,91_ = 14.6, *p* = 10^− 7^) (Fig. 1c) although a trend to significance was also observed in the right entorhinal cortex (Fig. 1d). Changes in miR-181c-5p levels were unrelated to volume variations of either hippocampus or parahippocampal gyrus. Neither did we find sex differences in regression coefficients for any of the correlations performed with mir-181c-5p and AD-related brain regions (Additional file 2: Figure S2C-H).

### Relationship between serum miR-181c-5p levels, cortical thickness, and cortical glucose consumption

**Fig. 2:**
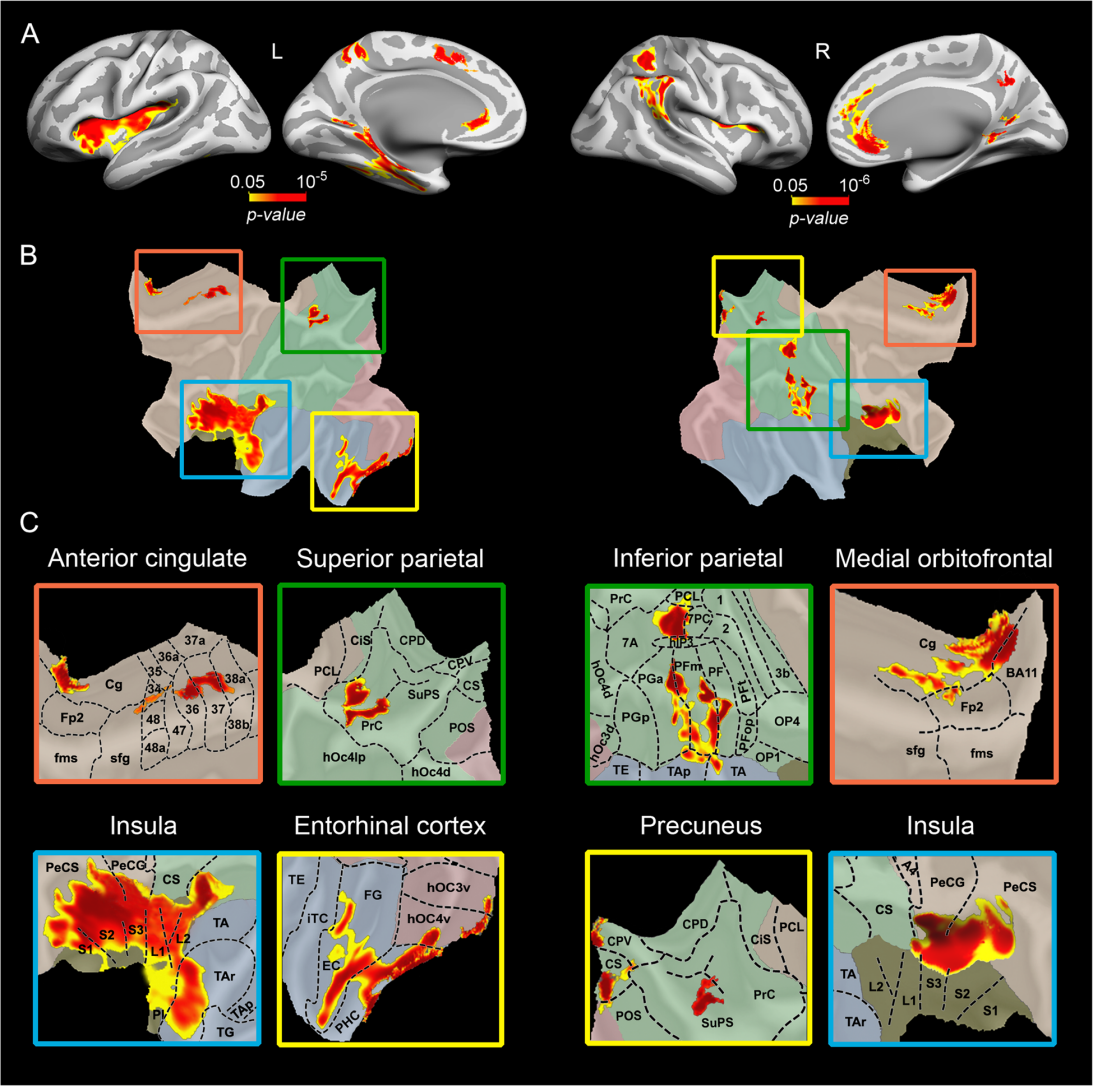
Vertex-wise regression analysis, adjusted by age and sex, to evaluate correlations between serum levels of miR-181c-5p and cortical glucose metabolism, measured with FDG-PET. **a** Significant patterns of correlations were represented on inflated cortical surfaces (L, left; R, right). Color bars represent corrected *p*-values (*p* < 0.05) using a hierarchical approach based on sequential statistical thresholding [35]. **b** Significant patterns of correlations displayed on flattened cortical surfaces. Squares with colored borders limit the location of significant regional changes. **c** The surface of the square was zoomed on flattened cortical maps displaying cytoarchitectonic delimitation of affected regions [36–40]

We next sought to investigate whether changes in serum levels of miR-181c-5p were correlated with variations in cortical glucose consumption, after removing the effects of age and sex. Results indicated that decreased serum expression of miR-181c-5p was associated with lower glucose consumption in superior parietal regions bilaterally (left: *p* = 10^− 4^; right: *p* = 10^− 6^), right inferior parietal areas (*p* = 10^− 6^), and right precuneus (*p* = 10^− 6^). Decreased FDG uptake within bilateral insula was also related to lower miR-181c-5p levels (left: *p* = 10^− 4^; right: *p* = 10^− 5^). Reduced serum levels of miR-181c-5p further predicted hypometabolism in the left entorhinal (*p* = 10^− 5^), right lingual gyrus (*p* = 10^− 4^), left superior frontal (*p* = 10^− 3^), and medial aspects of the right orbitofrontal cortex (*p* = 10^− 4^). These results are summarized in Table 2 and illustrated in Fig. 2. No significant associations were found between miR-181c-5p levels and changes in cortical thickness.

**Table 2:** Significant correlations between decreased serum levels of miR-181c-5p and lower cortical glucose uptake measured by FDG-PET

Cortical region	CS (mm^2^)	r	*P*
L insula	3891	0.29	10^−4^
L entorhinal	1588	0.3	10^−5^
L superior parietal	431	0.39	10^−4^
L superior frontal	245	0.31	10^−3^
L anterior cingulate	174	0.33	10^−4^
R inferior parietal	1531	0.38	10^−6^
R insula	839	0.37	10^−5^
R medial orbitofrontal	819	0.34	10^−4^
R superior parietal	583	0.35	10^−6^
R lingual gyrus	160	0.39	10^−4^
R precuneus	144	0.37	10^−6^

*CS* Cluster size; it refers to the extent of significant correlation between serum levels of miR-181c-5p and cortical glucose uptake. *L* Left and *R* Right cortical hemisphere. Regression analyses were adjusted by age and sex. r: Pearson correlation coefficient; p: exact *p*-value (corrected for multiple comparisons)

## Discussion

There is an urgent need for inexpensive and reliable biomarkers able to identify individuals at greatest risk of developing AD. While blood molecules are ideally suited for this endeavor [41], lack of standardization of pre-analytic conditions and poor reproducibility of results preclude their use as first-line diagnostic tools in clinical settings. miRNAs are stable circulating molecules that have shown to be deregulated in the prodromal and clinical phases of AD [42]. However, their usefulness for detecting at-risk subjects for developing AD remains to be determined. In the present study, we have shown that decreased serum expression of miR-181c-5p was associated with higher plasma Aβ_1–40_ levels, deficits in cortical glucose metabolism, and volume reduction of the entorhinal cortex in asymptomatic elderly subjects. Overall, these results are likely revealing aging-related cerebral vulnerability linked to altered expression of miR-181c-5p, which has previously been associated with Aβ regulation [19] and cerebral ischemia/hypoxia in both humans and animal models [43, 44], and has been found to be deregulated in the brain [21–23] and blood [12, 17, 24, 25] of AD patients.

Recent studies have assessed the role played by serum/plasma expression of miR-181c as AD biomarker. Most of them evaluated the miR-181c-5p [12, 17, 25], except for one that employed the miR-181c-3p [24]. Here we found that decreased serum levels of miR-181c-5p were associated with higher concentrations of plasma Aβ_1–40_. Previous studies have revealed that plasma Aβ_1–40_ is increased in familial AD with mutations in the genes encoding presenilin or the amyloid precursor protein [45, 46], in patients with Down’s syndrome [47, 48], and in first-degree relatives of AD patients who are at increased risk of developing the disease [49]. Moreover, elderly subjects with higher plasma Aβ_1–40_ levels have shown slower processing and poorer memory together with bilateral thinning of the prefrontal cortex compared with those who had lower Aβ_1–40_ levels, suggesting that higher plasma Aβ_1–40_ may signal accelerated aging [50]. Plasma Aβ_1–40_ levels have also shown to be elevated in cerebral small vessel disease, a frequent aging-related pathology that contributes to the development of dementia [51, 52]. Recent evidence indicated that miR-181c-5p is downregulated in plasma of acute stroke patients [43] and in the hippocampus of rat models of cerebral ischemia/hypoxia [44]. These studies have also shown that miR-181c agomir exacerbates brain ischemia-reperfusion injury [43] and improves cognitive deficits induced by chronic cerebral hypoperfusion [44]. Based on this evidence, we speculate that associations between miR-181c-5p and Aβ_1–40_ are likely instigated by chronic cerebral hypoperfusion and altered neurovascular coupling that lead to increased Aβ_1–40_ levels in aging conditions.

Contrary to what might be expected, especially considering previous evidence linking miR-181c to increased Aβ_1–42_ levels [20] and regulation of Aβ deposition in hippocampal cultures [19], we found no significant associations between serum mir-181c-5p and plasma Aβ_1–42_ concentrations. It may happen that the relationship between mir-181c-5p and Aβ_1–42_ is specifically linked to AD, whereas cerebral changes associated with decreased serum levels of mir-181c-5p is signaling aging-related cerebral vulnerability of unspecified origin. On the other hand, our results differ from those reported in [25]. Siedlecki-Wullich and colleagues [25] showed significant upregulation of miR-92a-3p, miR-181c-5p and miR-210-3p in the plasma of both MCI and AD subjects. In our study, performed in cognitively normal elderly subjects, we found significant associations between decreased serum expression of miR-181c and increased plasma levels of Aβ_1–40_, deficits in cortical glucose metabolism, and volume reduction of the entorhinal cortex. We speculate that both studies are likely revealing different phenomena (AD vs. aging-related cerebral vulnerability) associated with deregulation of miR-181c-5p. However, it should be mentioned that the two studies employed different blood compartments in miR-181c-5p experiments (serum vs. plasma). Although both plasma and serum are commonly used for extracellular miRNA detection, we selected serum to avoid sample hemolysis and to obtain higher miRNA concentrations [53].

To our knowledge, only a few studies have specifically evaluated the significance of combining serum expression of specific miRNAs and macroscopic brain changes as AD biomarkers. In a seminal work, Cheng and collaborators [54] identified an AD-specific serum 16-miRNA signature that was validated with amyloid-PET imaging, showing high accuracy during early- to mid-AD stage. Further research has revealed that associations between serum miR-223 and magnetic resonance spectroscopy markers of neuronal damage (i.e., N-acetylaspartate and myo-inositol) are able to predict AD severity through inflammatory and apoptosis pathways [55]. A recent study has also shown that lower serum expression of 5 AD-related miRNAs (miR-9-5p, miR-29b-3p, miR-34a-5p, miR-125b-5p, and miR-146a-5p) was associated with poorer cognitive functioning and structural and metabolic cortical deficits in aging, indicating that these miRNAs are biologically meaningful in senescence and may play a role as biomarkers of cerebral vulnerability in late life [29]. Since lower miR-181c expression has been tightly related to AD [19], our results linking decreased miR-181c-5p levels and atrophy of entorhinal cortex may be suggestive of accelerated aging and/or reveal certain susceptibility to developing AD. Further research is definitely warranted to clarify if the link between miR-181c-5p and entorhinal cortex may serve as a surrogate marker of AD vulnerability.

We have further shown that decreased serum expression of miR-181c-5p was associated with widespread cortical hypometabolism rather than with patterns of cortical thinning. Many studies have confirmed that reduced FDG-PET brain metabolism precedes cortical atrophy in early AD [56, 57]. In the present study, hypometabolic cortical regions that correlated with lower levels of miR-181c-5p are reminiscent of those that predict conversion to dementia in asymptomatic subjects (i.e., temporo-parietal and prefrontal cortex) [58], which may indicate certain susceptibility to developing AD in individuals with decreased miR-181c-5p. Moreover, previous studies have found that oxygen-glucose deprivation downregulates miR-181c expression in primary microglia by targeting Toll-like receptor 4 (TLR4), suggesting that miR-181c may also be involved in the regulation of the inflammatory response to hypoxic injuries [59]. Therefore, we speculate that, initially, early soluble Aβ oligomers lead to miR-181c deregulation [19] and subsequently lower miR-181c expression levels contribute to AD progression through deregulation of inflammatory response to hypoxia [59]. In this context, a deficient cortical glucose metabolism may reinforce multiple feedback loops of disease progression [60] accelerating the emergence of cognitive impairment. Alternatively, deregulation of miR-181c-5p may instigate the pathogenesis of AD by targeting specific proteins at the post-transcriptional level. This hypothesis is supported by experiments showing that low expression of miR-181c in the hippocampus of senescence-accelerated mice increases the collapsin response mediator protein-2 [61], a microtubule associated protein whose phosphorylation is required for Aβ-induced memory deficits [62]. Future experiments are required to determine whether miR-181c-5p deregulation is the cause or the consequence of cerebral vulnerability in aging and/or AD pathogenesis.

Accumulated research has highlighted the role played by serum miRNAs as potential biomarkers for AD [12–18]. In one of these studies, authors showed a significant upregulation of miR-455-3p in serum of AD patients, which was validated in postmortem AD brain tissues, AD cell lines, and in the cerebral cortex of amyloid precursor protein transgenic mice [18]. These findings have been recently extended to AD fibroblasts cells [63]. miR-455-3p was further related to genes directly associated with AD pathogenesis, revealing possible molecular links between miR-455-3p and AD progression [18, 63]. While these findings strongly suggest that miR-455-3p may be a potential serum biomarker for AD, its remains to be established its role in detecting asymptomatic subjects at risk for developing AD.

The present study is subject to certain limitations that should be mentioned. Participants were clinically normal elderly subjects. Therefore, our results do not allow establishing the prognostic value for the development of AD. Follow-up studies are necessary to determine to what extent lower serum expression of miR-181c-5p is a biomarker of AD, and whether cerebral markers reported here contribute to validate that prediction. Moreover, subjects lacked surrogate biomarkers to confirm AD pathology (e.g., CSF or PET-based biomarkers), impeding to determine the risk for AD. Consequently, these findings should be replicated in a well-characterized cohort with AD biomarkers and compared with other neurodegenerative diseases (e.g., vascular dementia, Parkinson disease, fronto-temporal dementia) to further establish their specificity in AD.

## Conclusions

In summary, our findings indicate that lower serum expression of miR-181c-5p is associated with higher plasma concentrations of Aβ_1–40_, widespread patterns of cortical hypometabolism, and volume loss of the entorhinal cortex in normal elderly subjects These results highlight the value of serum miR-181c-5p at determining cerebral vulnerability in normal aging.

## Supplementary information

**Additional file 1: Figure S1.** Mean/standard deviation of cycle threshold (Ct) values (3 replicas) of mir-181c-5p (gray circles) and cel-miR-39-3p (open circles) for each study participant.

**Additional file 2: Figure S2.** Correlations segregated by sex between serum levels of miR-181c-5p and plasma Aβ levels (A, B), and volume of AD-related brain regions (C-H). Variables included in the scatter plots correspond to the standardized residuals obtained from linear regression analyses adjusted by age, Aβ_1-42_ (in the case of Aβ_1-40_), Aβ_1-40_ (in the case of Aβ_1-42_), and ICV (in the case of left and right cerebral regions). Note that only correlations between serum mir-181c-5p and plasma Aβ_1-40_ levels yielded significant in women.

## Data Availability

The datasets used and analyzed in the current study are available from the corresponding author on reasonable request.

## Abbreviations

AD: Alzheimer’s disease
Aβ: Amyloid-β
BNT: Boston Naming Test
cDNA: Complementary deoxyribonucleic acid
CDR: Clinical Dementia Rating
CSF: Cerebrospinal fluid
Ct: Cycle threshold
FCSRT: Free and Cued Selective Reminding Test
FDG: 2-[18F]fluoro-2-deoxy-D-glucose
MFQ: Memory Functioning Questionnaire
miRNA: Micro ribonucleic acid
MMSE: Mini Mental State Examination
MRI: Magnetic resonance imaging
mRNA: Messenger ribonucleic acid
PET: Positron emission tomography
qRT-PCR: Quantitative real-time Polymerase Chain Reaction
ROI: Region of interest
RT: Reverse transcription
TLR4: Toll-like receptor 4
TOL: Tower of London
WM: White matter
