# Supplementary figures and images for: Lower serum expression of miR-181c-5p is associated with increased plasma levels of amyloid-beta 1–40 and cerebral vulnerability in normal aging
Source: Transl Neurodegener. 2019 Nov 4;8:34. doi: 10.1186/s40035-019-0174-8 (PMC6827222; doi:10.1186/s40035-019-0174-8)

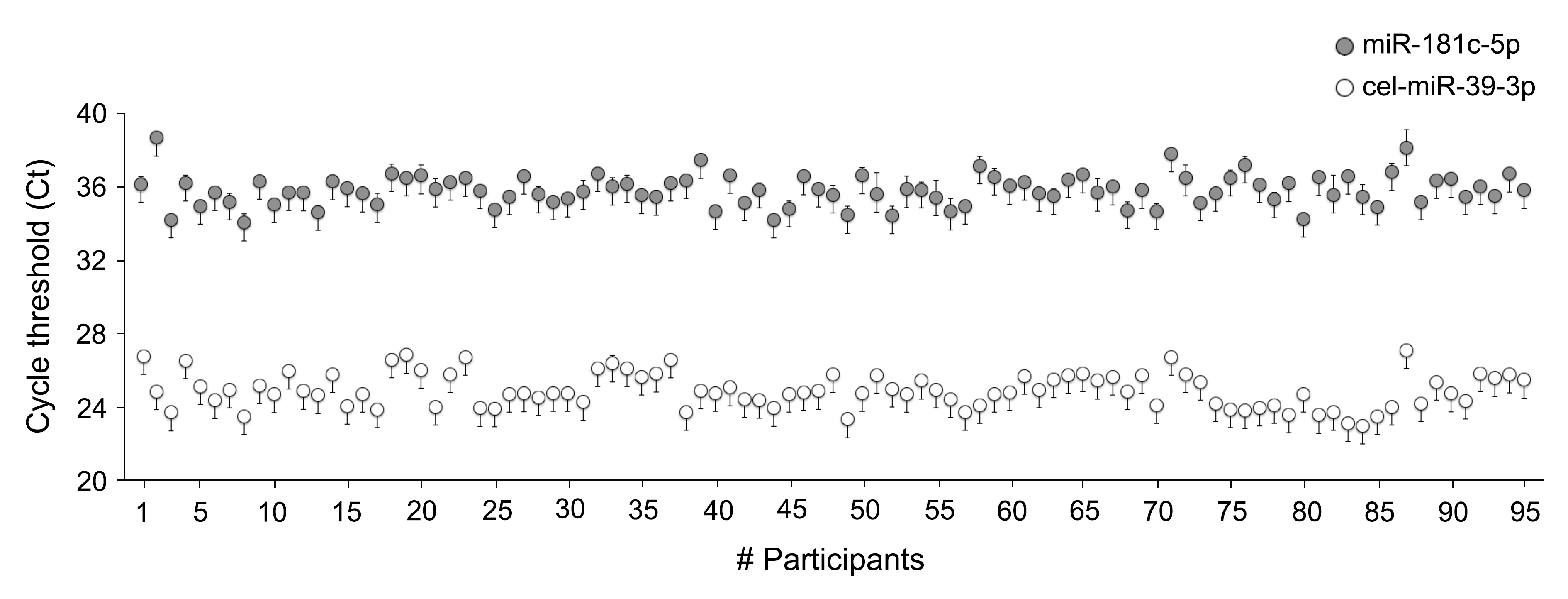

Supplement: Supplementary file 1 — Additional file 1: Figure S1. Mean/standard deviation of cycle threshold (Ct) values (3 replicas) of mir-181c-5p (gray circles) and cel-miR-39-3p (open circles) for each study participant. [file 40035_2019_174_MOESM1_ESM.tif]

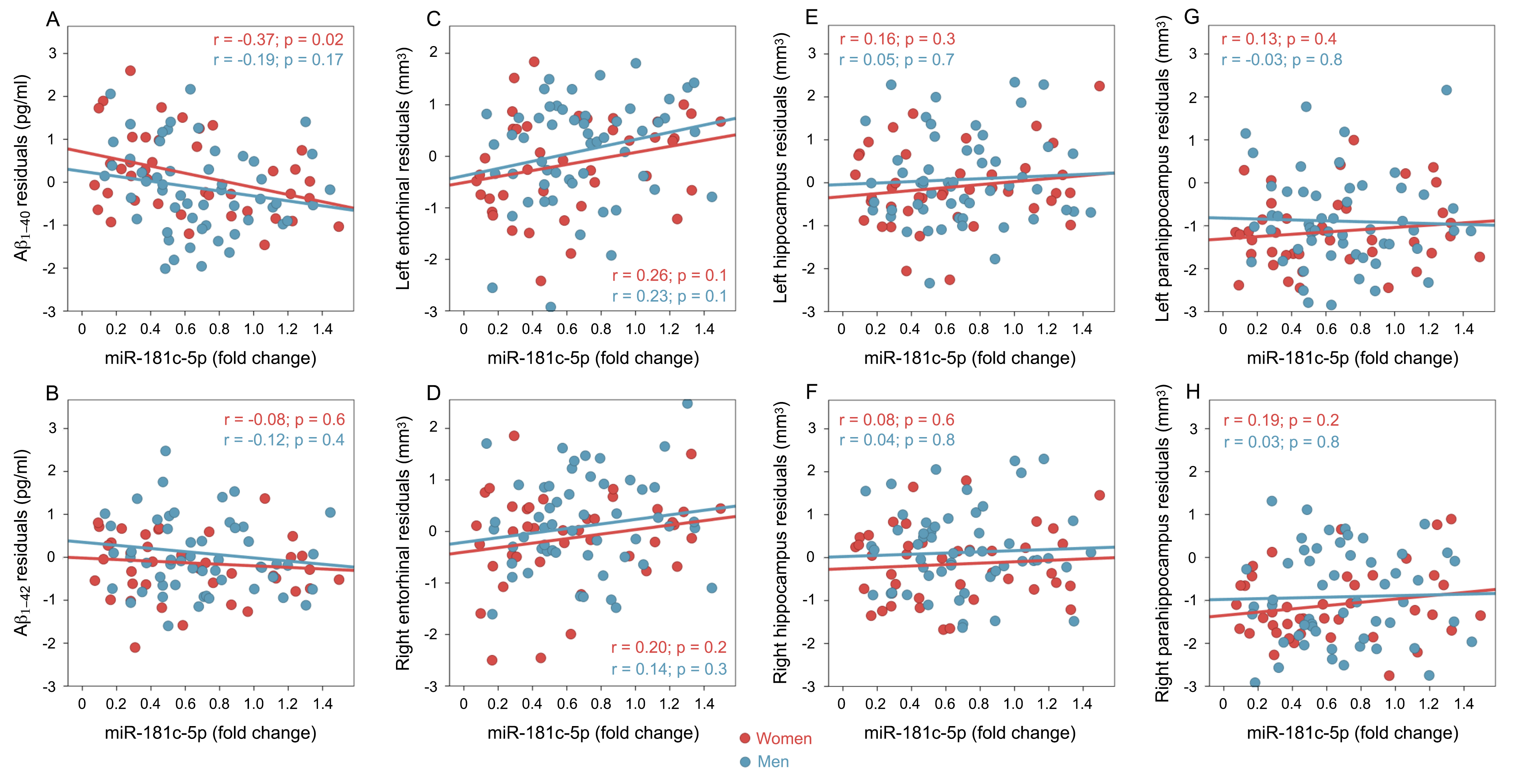

Supplement: Supplementary file 2 — Additional file 2: Figure S2. Correlations segregated by sex between serum levels of miR-181c-5p and plasma Aβ levels (A, B), and volume of AD-related brain regions (C-H). Variables included in the scatter plots correspond to the standardized residuals obtained from linear regression analyses adjusted by age, Aβ1-42 (in the case of Aβ1-40), Aβ1-40 (in the case of Aβ1-42), and ICV (in the case of left and right cerebral regions). Note that only correlations between serum mir-181c-5p and plasma Aβ1-40 levels yielded significant in women. [file 40035_2019_174_MOESM2_ESM.tif]
